# Effect of Body Mass Index on complications and success rates of percutaneous nephrolithotomy-A tertiary care hospital experience

**DOI:** 10.12669/pjms.38.8.3663

**Published:** 2022

**Authors:** Nadeem Iqbal, Aisha Hasan, Sajid Razzaq, Fatima Saqib Rashid

**Affiliations:** 1 Nadeem Iqbal, Shifa International Hospital, Islamabad, Pakistan; 2 Aisha Hasan, Shifa College of Medicine, Islamabad, Pakistan; 3 Sajid Razzaq, Sheikh Khalifa Bin Zayed Hospital/Poonch Medical College, Rawalakot, Azad Kashmir, Pakistan; 4 Fatima Saqib Rashid, Shifa College of Medicine, Islamabad, Pakistan

**Keywords:** PCNL, Calculi, Stone free rates, Body Mass Index, Complications

## Abstract

**Background and Objectives:**

Urolithiasis is prevalent globally. Over time, innovation in endoscopic instruments and miniaturization has untangled the interventional strategy for carrying out remedial surgical procedures for renal stones. However, studies have been scarce as for sequelae of Percutaneous Nephrolithotomy (PCNL) in varying body mass index group patients in the developing world. We aimed to report success rates and complications in different BMI groups.

**Methods:**

This was a retrospective study wherein data of 359 patients was reviewed in charts. These patients went through PCNL at our department from July 2011 till September 2019. Three groups of patients were made in agreement with WHO BMI classification. Information concerning study variables was noted in designated and then processed in SPSS version 16 for the statistical computations.

**Results:**

On the whole, the mean age of patients was 44.6± 14.4 years. While the mean calculus size was 3.1± 1.4 cm. Moreover, the majority of stones in all the three groups belonged to Guy’s stone score 1 and 2 (see Table-I). The overall mean procedure time and inpatient stay were almost comparable in the obese group. The highest stone-free rate was observed in the normal weight group (77.69 %), however, stone-free status in overweight and obese groups was not comparatively too lower (p=0.74). Complication rates were being close among the three groups.

**Conclusion:**

PCNL can be ventured with safety and in an effectual manner for attaining stone treatment goals alike in obese and non-obese group patients.

## INTRODUCTION

It is known that individuals having higher body mass index (BMI) are at an expanded peril of cardiovascular problems, metabolic aberration, malignancies, and untimely death.^1^,^2^ It’s also a fact that becoming fatter has been linked to increased likelihood of nephrolithiasis.^1^,^3^ Besides these, various scholarly works have also exhibited obesity to be an important independent risk factor for surgical and anesthetic challenges such as atelectasis, thromboembolic emergencies and wound problems.^3^-^7^ Surgeons must select patients justly. This necessitates risk and end results data into all kinds of surgical approaches in patients of different BMI categories.^5^-^7^

Extracorporeal shock-wave lithotripsy (ESWL) is being considered a preferred modality to manage minute and moderate-sized renal stones; however, this option is under productive in obese patients.^5^-^8^ Renal stones having a size of more than 2 cm or those who are not aspirants for EWSL, or those having previously failed ESWL can be better treated with percutaneous nephrolithotomy (PCNL). So due to the diminished benefit of ESWL in obese patients, PCNL has become well-liked by urologists while dealing renal calculi in those with raised BMI. Even though PCNL is a better option than ESWL in obese people, but it’s pertinent here that it still poses some of the treatment challenges in obese people taking into account the difficulty in enduring prone positioning. Moreover, hefty subcutaneous fat covering can forge the nephroscope too short.^6^-^8^ In the past, there have been some studies analyzing the benefit and safeness of PCNL in obese and morbidly obese persons, but their results are meagre and exhibits varied results.^8^ These results in obese patients have remained controversial.

Many urologists feel hesitation while they contemplate procedure in those with exalted BMI. However, as obesity trending ratios are on the rise globally, the superfluity of stone disease is also anticipated to jump up further. So we need to take the challenge of doing PCNL in obese patients as well.^8^-^11^ That’s the motive behind this study wherein we compared sequel and adverse net results of PCNL among those with varying BMI with a motive to ascertain the safeness and stone-free rates of this procedure in those with above normal BMI.

## METHODS

Data of 359 patients was reviewed in charts. These patients underwent PCNL at our department from to July 2011 to September 2019. The aim of study was to draw comparison of PCNL outcomes among different groups of BMI, in regards to their total operative time stone clearance, post-operative complications and hospital stay. Informed consent was acquired from all patients prior the procedure and they were counselled regarding possible outcomes and complications. Patients of age less than 18 years, those having abnormal coagulation profile, active urinary tract infection, subjects having congenital renal anomaly, those who had prior history of ipsilateral renal surgery or prior sessions of shock wave lithotripsy and those who failed to come for follow up were excluded from the study. Additionally, those patients whose CT scan studies were not available prior to surgery had to be excluded.

Three groups of patients were made in agreement with WHO BMI classification. Group-1 comprised of subjects having a normal BMI (18 to 24.9 kg/m2 range); Group-2 consisted of patients who were overweight (BMI range 25 to 29.9 kg/m2); Group 3 incorporated patients who were obese (BMI in range of 30 to 34.9 kg/m2).

Patient demographic information was recorded by residents at the department, which included patient age, gender, past surgical history for stone disease, body mass index (BMI). Stone characteristics were also included such as size of stone in cm, location of stone and number of stone present. Stone size was assessed as the diameters (cm) on CT scan images. The total stone bulk was grand total of individual length of multiple stones calculated on CT scan images. Post-operative variables recorded comprised of hemoglobin drop after the procedure, demand for the analgesics, recording of procedure related complications (modified clavian classification), date of discharge from hospital, residual stones status on post procedure X-ray KUB/ Ultrasound KUB (Kidney+Ureter+Bladder).

Once surgical option of doing percutaneous nephrolithotomy (PCNL) was pursued, blood investigations consisting of complete blood count, renal functions (serum urea, blood urea nitrogen and creatinine), electrolytes and coagulation tests were done prior to the time of inpatient admission. After proceeding with blood grouping and cross matching arrangement for blood was requested. Relevant antibiotic treatment with antibiotics was administered to patients, who were positive for bacterial colonies on urine cultures preoperatively.

### PCNL Procedure:

After taking the informed consent, patient was shifted to Operation Theater. WHO operation theatre safety check list was adhered to before starting the procedure. After inducing the general anesthesia, the patient was laid down in a lithotomy position, cleaned and draped. Then an open-end ureter catheter (size 6 Fr) was inserted into the renal pelvis with the assistance of fluoroscopy is advanced up until the renal pelvis. Then it was secured along with a Foleys catheter. Then patient was positioned in prone position. Triangular or Bull’s eye technique was utilized according to case to case. Lower pole was preferred to get an entry to the calyx. After that, metallic alken dilators were used serially up to 27 Fr. Then an Amplatz sheath (size=30 Fr) was made to glide over these metallic dilators into the pelvicalyceal system. In next step, a rigid nephroscope of size 24 Fr was guided into the renal collecting system with aid of a camera vision; stone was identified and a pneumatic lithoclast was introduced to break the stone into pieces. These broken stone fragments were brought out with the help of three prong stone graspers. Another glide wire was thrusted through the open end in a retrograde fashion. After securing this glide wire, open end was pulled back down wards and a stent was passed under nephroscope vision (size of stent =6 Fr, 26 cm length). Finally, in case of necessity for placing a nephrostomy tube, a 20 Fr tube was kept in place and connected to a collecting bag. The nephrostomy tube was secured with silk thread. In case of absence of flank pain and peri tube leaking; once the nephrostomy tube was clamped; it was removed.

### Follow up of patients:

Complications were recorded in line with the Modified Clavien system. The patients were subjected to post op follow up investigations at one and three months out door with Ultrasound and X-Ray KUB to look for status of any residual stones. The existence of residual fragments of size ≤ 4 mm or absence of any stone was documented as success of therapy.

### Statistical Analysis:

Data was gathered and arranged in the proformas by the urology resident and then entered the information in the statistical analysis software file. Analysis was attained by utilizing SPSS version 16. Implementation of Mean along with standard deviation values was utilized in case of the continuous variables .While frequency/percentages represented categorical factors. We availed ANOVA test for comparing the continuous factors and Chi-square test was utilized to weigh up categorical values between the groups. A p-value of <0.05 was judged as statistically crucial value.

## RESULTS

In entirety 359 subjects were incorporated in the final analysis. There were 130 patients in Group 1 that comprised of subjects having a normal BMI (18 to 24.9 kg/m2 range); 145 patients in Group 2 that consisted of patients who were overweight (BMI range 25 to 29.9 kg/m2); while Group 3 had 84 patients who were obese (BMI in range of 30 to 34.9 kg/m2).

On the whole, the mean age of patients was 44.6± 14.4 years. While the mean calculus size was 3.1± 1.4 cm. However, there was no significant difference between the three groups in terms of age and stone size (Table-I).One hundred and fifty three procedures were done on the right side (42.6%), while 206 (57.4%) patients had it on the left side. Although the ratio of right-sided PCNL was relatively more frequent in the obese group (Group-3) as compared to other groups, it was statistically insignificant, p=0.11 (Table-I). Moreover, majority of stones in all the three groups belonged to Guy’s stone score 1 and 2 (Table-I). The complexity of stones formulated on Guy’s stone score was identical among the three groups (P=0.53).

**Table-I:** Demographic variables.

	Normal Weight	Over Weight	Obese	P-value
Number	130	145	84	---
Mean Age	43.06±16.67 years	46.24±13.33 years	44.43±12.59 years	0.19
Male	93 (71.53%)	109 (75.12%)	51 (60.71%)	0.06
Female	37 (28.46%)	36 (24.82%)	33 (39.28%)	
Right Renal stone	52 (40%)	57 (39.31%)	44 (52.38%)	0.11
Left Renal stone	78 (60%)	88 (60.68%)	40 (47.61%)	
Body Mass Index	22.01±1.87 Kg/m^2^	26.94±2.23 Kg/m^2^	32.64±4.08 Kg/m^2^	<0.0001
Mean stone size (cm)	3.17±1.61 cm	3.08±1.24 cm	3.12±1.58 cm	0.87
***Guys Stone Score***				
Guys Stone Score 1	93 (71.53%)	104 (71.72%)	64 (76.19%)	0.53
Guys Stone Score 2	33 (25.38%)	30 (20.68%)	17 (20.23%)	
Guys Stone Score 3	1 (0.76%)	5 (3.44%)	2 (2.38%)	
Guys Stone Score 4	3 (2.30%)	6 (4.13%)	1 (1.19%)	

The overall mean procedure time was not much dissimilar among the groups and hence being statistically insignificant (Table-II). The inpatient stay was almost comparable in the obese group when compared to the normal weight and overweight groups (Table-II, it is statistically not significant).While mean analgesic doses were also not significantly different among the three groups (Table-II). The highest stone-free rate was observed in the normal weight group (77.69 %), however, stone-free status in overweight and obese groups was not comparatively too lower (p=0.74) as can be seen in Table-II.

**Table-II:** Details of Procedure Outcomes.

	Normal (n=130)	Over weight (n=145)	Obese (n=84)	P-value
Stone free rate	101 (77.69 %)	107 (73.79 %)	63 (75 %)	0.74
Residual stones	29 (22.31%)	38 (26.21%)	21 (25%)	0.74
Mean Operative time	142.54±64.05 minutes	146.89±78.10 minutes	139.69±53.85 minutes	0.72
Hospital stay	3.46±1.27 days	3.24±1.35 days	3.43±1.20 days	0.41
Analgesic doses	2.25±1.89	2.57±1.73	2.77±2.01	0.11

Complications were described in accordance with Clavien-Dindo Classification. It is obvious from Table-III that ileus (without need NG tube), fever and transient hematuria were almost similarly encountered among the three groups (Clavien Grade-1 complications). Interestingly, transfusion rates were not different among these groups (Table-III) and statistically the difference was insignificant (p=0.73). One patient (0.68%) had persistent hematuria needing repeated bladder washing in Group-II (overweight group). He needed repeat in-house admission and underwent a CT scan with contrast that revealed renal vascular injury on the operated side. He was successfully managed with renal artery angioembolisation (Table-III). The sepsis rate was not much varying among the different BMI groups (Table-III). Major Complications (Clavien Grade 3 and 4) such as need for nephrectomy, bowel injury and death were not encountered in any of the BMI group (Table-III).

**Table-III:** Complications.

Complication grade	Complication name	Normal (n=130)	Overweight (n=145)	Obese (n=84)	p-value
1	Fever	3 (2.30%)	6 (4.13%)	3 (3.57%)	0.69
1	Illeus without need NG tube	1/ (0.76%)	0/51 (0%)	1 (1.19%)	0.46
1	Pelvicalyceal puncture (extravasation)	2 (1.53%)	1 (0.68%)	2 (2.38%)	0.56
1	Transient hematuria	13 (10%)	16 (11.03%)	9 (10.71%)	0.96
2	Transfusion	7 (5.38%)	5 (3.44%)	4 (4.76%)	0.73
2	Sepsis	6 (4.61%)	6 (4.13%)	3 (3.57%)	0.93
3	Peri nephric collection/abscess	6 (4.61%)	7 (4.82%)	6 (7.14%)	0.68
3	Bowel injury	0%	0%	0%	---
3	Renal vascular injury requiring angioembolisation	0%	1 (0.68%)	0%	0.47
4	Septic Shock ICU manage	0 %	0%	0%	---
5	Death	0 %	0%	0%	---

## DISCUSSION

It has been estimated that the lifetime probability to bear a kidney stone reaches around 12%.^12^ Pakistan is situated in the stone zone which means that an exorbitant frequency of renal stones is encountered in Pakistan.^12^-^13^ In the early days of renal stone treatment, open surgery was the only choice, but as time passed and technological advancement took place then minimally invasive, endoscopic procedures and extra-corporeal shock wave lithotripsy (ESWL) were introduced which then nearly superseded the previously established open surgery for renal stones treatment.

In the modern society, an increasingly inactive way of life and diet enriched in fats have been on the rise. These lifestyle changes have resulted in obesity. It is said that in the USA, approximately one-third of adults were obese in the period 2011–2012.^14^ Obesity is rampant in he developing nations, including Pakistan. There is a profound rise in the prevalence of obesity; recent data have suggested that nearly one billion or more adults are categorized as overweight, and at least 300 million of them are said to be clinically obese.^15^

Obesity is hypothesized to be related to surgical and anesthetic complications leading to higher morbidity.^16^-^17^ Anesthesia can be a challenging task in obese patients because of difficult intubation, a diminished total lung capacity in the prone positioning for PCNL leading to complications. In some centers, the supine position for PCNL has been tried to reduce the possible complications, however, it cannot be done in every center and in every patient.^17^-^18^ The per-operative challenge is the thicker layer of subcutaneous fat that renders the nephroscope too short, adding to the difficulty faced by the operating surgeon during the procedure, longer operative time, morbidity, risk of complications, longer hospital stay, inferior stone clearance rates, and economic burden on patients. To date, there is a paucity of literature in the developing world as to how BMI can affect the results of PCNL as to stone-free rates and complications. In this study we have compared operative time, hospital stay, stone clearance rates, and postoperative complications in patients undergoing PCNL procedure in different BMI groups.

In a study by fahad et al it was found that there was no difference in blood loss or operative time and the hospital stay when different BMI groups’ patients were compared for the outcomes.^19^ They had a small sample size of 114 patients. In yet another study by Akbulut et al BMI values out of 182 patients, 49 had subjects had BMI values above 30 kg/m2. They did not find any difference among these groups in terms of operation duration in minutes, per-operative fluoroscopy duration, a stretch of inpatient hospital stay, drop in hemoglobin, and overall complication rates.^20^ Most importantly, the stone-free rates of 70.7% and 71.4% were noted in the two groups.^20^ In present study, the highest stone-free rate was observed in the normal weight group (77.69%), however, stone-free status in overweight and obese groups was not comparatively too lower (p=0.74) as can be seen in Table-II. Akbulut et al had lesser stone free rates as compared to our despite the fact that stone size in our study was greater as compared to theirs. This may be due to different expertise level in different centers.^20^ Şimşek et al deduced from their study that percutaneous nephrolithotomy was a safe and effective way of treating renal stones. Additionally, body mass index does not affect success rates or the frequency of complications post-PCNLl.^21^ In present study, ileus (without need NG tube), fever and transient hematuria were almost similarly encountered among the three groups (Clavien grade 1 complications). Interestingly, transfusion rates were not dissimilar among these groups (see Table-III) and statistically the difference was insignificant (p=0.73).

In a study by Cemal et al patients were divided into two groups: BMI < 30 kg/m2 (non-obese group) and BMI ≥ 30 kg/ m2 (obese group). They noted mean operation duration as 72.4±3.81 minutes and 65.4±2.75 minutes in the non-obese and the obese groups respectively. Moreover, they noted a mean duration of fluoroscopy time of 149.3±6.06 seconds and 144.4±9.74 seconds in these groups respectively (statistically not significant). Similarly, No notable difference was observed between these BMI groups, with regards to the intraoperative and post-operative need for blood transfusion.^23^ In present study, the complexity of stones formulated on Guy’s stone score was identical among the three groups (P=0.53). The overall mean procedure time was not much dissimilar among the groups and hence being statistically insignificant (Table-II). The inpatient stay was almost comparable in the obese group when compared to the normal weight and overweight groups (Table-II, it is statistically not significant).While mean analgesic doses were also not significantly different among the three groups (Table-II).

Proper percutaneous access in an obese patient is an utmost challenge because of the superfluous soft tissue which reduces the image quality during fluoroscopy and hence the accurate identification of a target calyx. Secondly, in an obese person, skin-stone distance is more hence rendering the access to the collecting system and dilatation of the tract more difficult. Thirdly, there are limitations of the length of the nephroscope and instruments.^22^-^24^ However, there are some tricks for tackling these issues such as the choice of the shorter length of the access track to allow ease of instrument maneuverability during the procedure. Furthermore, extra-long custom-trimmed access sheaths may help in gaining proper access tract. Alternatively, placing two access sheaths in series can cover the length of the access tract .along the tract. Utilization of flexible nephroscope or widely incised skin and subcutaneous tissue deeper down to the muscular fascia can help in shortening the tract length and simplify the percutaneous access.^24^ There is substantial exposure to radiation in obese patients because of enhanced delivery of radiation by the fluoroscopic devices to permit adequate imaging. Therefore, cutting down the quantity of fluoroscopy is called for. Pulsed fluoroscopic imaging can be used to curb radiation exposure in such patients.^25^

### Limitations of the study:

It includes the retrospective design of the study and the involvement of multiple surgeons in place of a single surgeon. The strengths of this study was due to the fact that complexity of stones formulated on Guy’s stone score was identical among the three groups. Such stone complexity categorization based on Guys stone score have not been clearly taken into account in previous studies regarding the subject matter. Multicenter prospective studies are lacking and hence should be pursued in future.

## CONCLUSION

PCNL can be ventured with safety and in an effectual manner for attaining comparable stone free rates and complications rates in obese patients and overweight subjects.

### Author’s Contribution:

**NI, SR:** Conceived, designed and did statistical analysis & editing of manuscript, is responsible for integrity of research. **NI, SR, FS, AH:** Did data collection and manuscript writing **NI, SR, FS, AH:** Did review and final approval of manuscript.

**NI:** Responsible for accuracy or integrity of the work.
